# Leptin receptor polymorphism increases the risk of painful symptoms in Brazilian women with endometriosis

**DOI:** 10.61622/rbgo/2025rbgo43

**Published:** 2025-07-15

**Authors:** Jéssica Vilarinho Cardoso, Daniel Escorsim Machado, Fernanda Nunes de Almeida, Plínio Tostes Berardo, Rui Medeiros, Jamila Alessandra Perini

**Affiliations:** 1 Universidade do Estado do Rio de Janeiro Rio de Janeiro RJ Brazil Universidade do Estado do Rio de Janeiro, Rio de Janeiro, RJ, Brazil.; 2 Fundação Oswaldo Cruz Escola Nacional de Saúde Pública Sergio Arouca Rio de Janeiro RJ Brazil Escola Nacional de Saúde Pública Sergio Arouca, Fundação Oswaldo Cruz, Rio de Janeiro, RJ, Brazil.; 3 Hospital Federal dos Servidores do Estado Rio de Janeiro RJ Brazil Hospital Federal dos Servidores do Estado, Rio de Janeiro, RJ, Brazil.; 4 Instituto de Educação Médica Rio de Janeiro RJ Brazil Instituto de Educação Médica, Rio de Janeiro, RJ, Brazil.; 5 Instituto Português de Oncologia do Porto Porto Portugal Instituto Português de Oncologia do Porto, Porto, Portugal.

**Keywords:** Endometriosis, Dysmenorrhea, Dyspareunia, Alelles, Polymorphism, genetic, Genotype, Receptors, leptin, Inflammatory cytokines, Pelvic pain

## Abstract

**Objective:**

Endometriosis pain is associated with inflammatory cytokines, such as leptin (LEP), through activation with its receptor (LEPR), and its expression can be influenced by the presence of genetic polymorphisms. Therefore, this study aims to evaluate the role of the *LEP* rs7799039 and *LEPR* rs1137100 polymorphisms in the painful symptoms of endometriosis in Brazilian women.

**Methods:**

A retrospective study was carried out in two Brazilian public hospitals with 237 cases of endometriosis, divided into two comparison groups according to the painful symptoms associated with the disease (absence or presence of severe and disabling symptoms). Genetic analysis was performed by real-time PCR technique, and association analyses were estimated using odds ratio (OR) and 95% confidence interval (CI), using a non-conditional logistic regression model.

**Results:**

Endometriosis cases showed a high prevalence of painful symptoms: 82% dysmenorrhea, 67% dyspareunia, 53% chronic pelvic pain, and 52% cyclical intestinal and 25% urinary complaints. Regarding genetic analyses, cases had 32.7% of the A allele and 11.4% of the AA genotype for the *LEP* rs7799039 G>A SNP, and 17.5% of the G allele and 2.5% for of GG genotype for the *LEPR* rs1137100 A>G SNP. There is a significant association of the *LEPR* rs1137100 polymorphism with chronic pelvic pain (OR=1.75; CI 95%=1.05-2.89) and dyspareunia (OR=1.78; CI 95%=1.01-3.12) in women with endometriosis.

**Conclusion:**

Our findings suggest that the *LEPR* rs1137100 polymorphism is associated with increased endometriosis-related gynecological pain and may be a potential target for molecular diagnosis of the disease and development of individualized treatment strategies.

## Introduction

Endometriosis is an estrogen-dependent disease that affects approximately 3 to 20% of women of reproductive age and is characterized by the presence of functional endometrial implants outside the uterine cavity.^(1)^ The occurrence of painful symptoms, impaired fertility, the invasive diagnostic process, and the various treatments that these women undergo have a negative impact on their physical, mental, and social well-being.^(1)^

The molecular mechanisms involved in the endometriosis are not fully understood; however, there is evidence that leptin (LEP) and its receptor (LEPR) levels are significantly elevated in the peritoneal fluid of patients with endometriosis, suggesting that this hormone may be associated with the pathogenesis of the disease.^(1-3)^ In addition, elevated levels of this hormone may activate signaling pathways that induce proliferation, inflammation, and angiogenesis, which are fundamental and well-known features of endometriosis and its painful symptoms.^(2)^

Genetic variants, such as single nucleotide polymorphisms (SNPs), in genes involved in the endocrine pathway can promote functional changes in their proteins, influencing disease development.^(4,5)^ The genes that encode leptin (LEP) and its receptor (LEPR) present two SNPs that stand out, *LEP* −2548G>A (rs7799039) and *LEPR* 326A>G (K109R, rs1137100), mainly because of their location in the regulatory and coding regions, respectively, which can affect the level of expression and transcription of their proteins and, consequently, influence the development of endometriosis.^(4,5)^ Therefore, the association of these SNPs with the prevalence of painful symptoms of endometriosis was investigated to help understand the molecular mechanisms involved in the disease and to aid in its diagnosis and treatment.

## Methods

A retrospective cross-sectional study was performed on 237 women with endometriosis diagnosed surgically by laparoscopy (n = 107) or laparotomy (n = 39) or after diagnosis of deep infiltrating endometriosis (DIE) by magnetic resonance imaging (MRI) (n = 91). Cases with visible ectopic implants were classified as having superficial endometriosis (SUP), ovarian endometrioma (OMA), or DIE. Women with peritoneal and ovarian lesions were considered to have OMA, and peritoneal and ovarian lesions associated with infiltrative endometriosis were considered to have DIE.^(6)^ Moreover, surgically diagnosed cases were stratified into stages I–II and III–IV as described in our previous study.^(7)^ Women with a prior history of cancer, chronic kidney disease related to hypertension, rheumatoid arthritis, or adenomyosis were excluded from this study.

Endometriosis-related symptoms were classified into four subtypes: mild, moderate, severe or disabling. Mild symptoms were those for which the woman did not need medication to control them. Moderate symptoms were those where medication taken at home controlled the pain. Severe symptoms were those in which the woman needed medication administered in a hospital setting, but this was unsuccessful, and disabling symptoms prevented them from carrying out their daily activities, as suggested by a previous study.^(8)^ Therefore, in the current study, severe and disabling symptoms of dysmenorrhoea, chronic pelvic pain, dyspareunia and cyclical bowel and urinary symptoms were evaluated as the "presence of endometriosis-related symptoms group" and compared with women with endometriosis without severe and disabling symptoms (absence of endometriosis-related symptoms group). Patients with mild and moderate symptoms or missing data on any of the endometriosis symptoms were not included in this analysis of the association of polymorphisms with endometriosis-related symptoms only.

Each participant's DNA from the whole blood sample collected in tubes containing EDTA was extracted following the manufacturer's instructions. Genotyping of the *LEP* rs7799039 and *LEPR* rs1137100 polymorphisms was performed using specific TaqMan probes (C_1328079_10 and C_518168_20, respectively) by Real-Time PCR technique (Applied Biosystems, Foster City, CA, EUA). The PCR conditions were described previously.^(7)^

The sample calculation was performed using the Epi Info 7 program, version 7.1.3 (http://wwwn.cdc.gov/epiinfo/html/downloads.htm), to detect a difference between the groups studied (absence and presence of symptoms), assuming a type I error power of 0.8 and 5%. From this, at least 110 women in each group should have been recruited. Continuous variables were presented as mean ± standard deviation and categorical variables were presented as percentage.

SNPs were tested for Hardy-Weinberg equilibrium (HWE) using the chi-square goodness-of-fit test. When the SNP had <10 women as variant homozygotes, these were combined with the heterozygotes to compare no variant allele versus at least one variant allele (dominant model). Differences in allelic and genotypic distribution were compared using Person's chi-square test. The risk association for symptoms of endometriosis was estimated using odds ratios (OR) with their respective 95% confidence intervals (95% CI), adjusted for confounding factors (age and BMI). All data analyzes were performed with SPSS^®^ statistics software (version 20.0), and a *P* value ≤0.05 was considered statistically significant.

All patients involved were recruited from two reference hospitals in Rio de Janeiro between 2011 and 2018 (*Hospital Federal dos Servidores do Estado* and *Hospital Moncorvo Filho*), with written informed consent and approval from the Human Research Ethics Committee of both institutions (HFSE 414/2011 and HMF 1.244.294/2015), as described in a previous study.^(7)^ This work has been carried out in accordance with The Code of Ethics of the World Medical Association (Declaration of Helsinki) for experiments involving humans.

## Results

**Figure 1:**
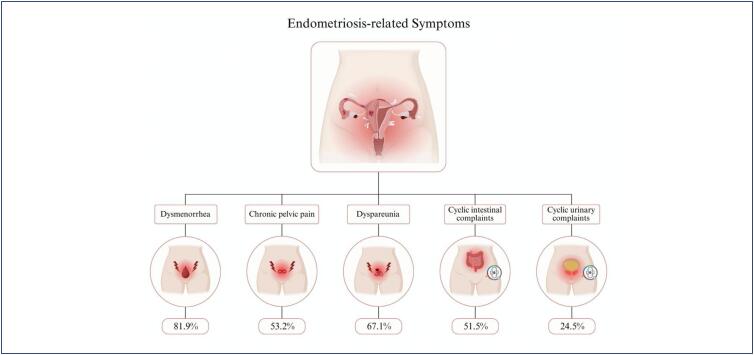
Frequency of painful symptoms related to endometriosis. The same woman can have more than one symptom. The mean time from symptoms onset to clinical diagnosis in the recruiting hospitals was 4.5 ± 6.5 years

Chart 1 describes the demographic and clinical characteristics of the study population. Most women were between 30-39 years old, had a normal BMI, and presented advanced stage and infiltrative lesions. The endometriosis cases exhibited a high prevalence of dysmenorrhea 82% (n=194), dyspareunia 67% (n=155), chronic pelvic pain 53% (n=126), and cyclical intestinal 52% (n=118) and urinary 25% (n=56) symptoms, according to the results of a previous study (Figure 1).

**Chart 1:** Demographic and clinical characteristics of the cases with endometriosis

Variables		Cases n(%)
**Age (Year)**		
	18 - 29	45(19.2)
	30 - 39	114(48.7)
	≥40	75(32.1)
	Missing	3
	
**Endometriosis classification**		
	SUP	19(8.0)
	OMA	61(25.7)
	DIE	157(66.3)
**BMI (Kg/m^2^)**		
	≤ 18.4	12(5.2)
	18.5 - 24.9	87(37.3)
	25 - 29.9	74(31.8)
	30 - 39.9	57(24.5)
	≥40	3(1.3)
	Missing	4
**Endometriosis stages** *		
	I-II	60(41.1)
	III-IV	86(58.9)

BMI - body mass index; SUP = superficial endometriosis; OMA - ovarian endometrioma; DIE -deep infiltrating endometriosis;

* Patients who were diagnosed by magnetic resonance imaging (n=91) did not have surgical staging of endometriosis

**Figure 2:**
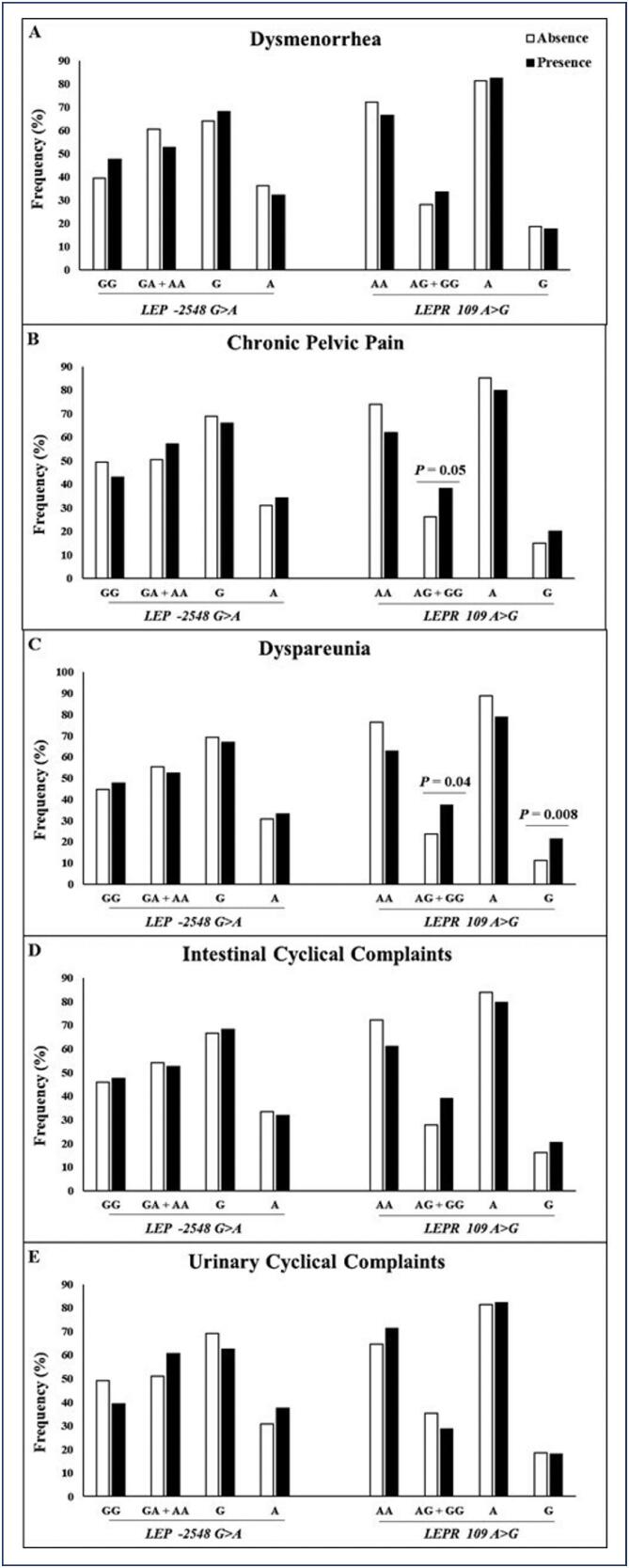
Genotypic and allelic frequencies of the *LEP* −2548G>A (rs7799039) and *LEPR* 109A>G (rs1137100) polymorphisms for the symptoms dysmenorrhea (A), chronic pelvic pain (B), dyspareunia (C), intestinal (D) and urinary cyclical complaints (E) among cases of endometriosis. Group of "absence" are patients without endometriosis-related symptoms. Group of "presence" are patients with severe and disabling symptoms endometriosis-related symptoms

The allele and genotypes distribution frequencies for the *LEP* rs7799039 G>A SNP were 32.7% (n=155) for the A allele, 42.6% (n=101) for the GA genotype and 11.4% (n=27) for the AA genotype, and for the *LEPR* rs1137100 A>G SNP were 17.5% (n=83) for the G allele, 30% (n=71) for the AG genotype and 2.5% (n=6) for the GG genotype. In this work, it was evaluated whether the studied SNPs were associated with the symptoms of endometriosis. Figure 2 shows the allelic and genotypic frequencies of the *LEP* and *LEPR* SNPs for each symptom among endometriosis cases. Women with chronic pelvic pain and dyspareunia had a higher frequency of the variant genotype (AG+GG) and allele (G) of the *LEPR* rs1137100 SNP compared to women without pelvic pain and dyspareunia. While for the *LEP* rs7799039 SNP, no significant differences in the prevalence of symptoms were found.

Based on the differences found for the *LEPR* rs1137100 SNP and the symptoms of chronic pelvic pain and dyspareunia, an association analysis of this SNP between the absence or presence of these symptoms was carried out, as shown in table 1. Both symptoms were positively associated with the *LEPR* SNP, demonstrating that the presence of at least one variant allele (*LEPR* G and AG + GG) led to a greater chance of developing these symptoms in women with endometriosis after adjusted analysis (age and BMI). Therefore, it is important to investigate this hypothesis in other populations, as there are no data in the current literature on the association of these SNPs with the presence of painful symptoms in endometriosis.

**Table 1:** Association analysis of the *LEPR* rs1137100 polymorphism in cases of endometriosis related to chronic pelvic pain and dyspareunia

LEPR rs1137100 A>G	Absence	Presence	p-value*	ORc (CI 95%)	p-value*	ORa (CI 95%)
**Chronic pelvic pain**						
Genotypes	n=111(%)	n=126(%)				
AA	82(73.9)	78(61.9)		1**		1**
AG + GG	29(26.1)	48(38.1)	0.05	1.74 (1.00 - 3.03)	0.01	2.07 (1.16 - 3.70)
Alleles						
A	189(85.1)	201(79.8)		1**		1**
G	33(14.9)	51(20.2)	0.13	1.45 (0.90 - 2.35)	0.02	1.75 (1.05 - 2.89)
**Dyspareunia** ***						
Genotypes	n=76(%)	n=155(%)				
AA	58(76.3)	97(62.6)		1**		1**
AG + GG	18(23.7)	58(37.4)	0.04	1.93 (1.04 - 3.58)	0.04	1.98 (1.05 - 3.74)
Alleles						
A	135(88.8)	244(78.7)		1**		1**
G	17(11.2)	66(21.3)	0.008	2.15 (1.21 - 3.81)	0.04	1.78 (1.01 - 3.12)

Group of "absence" are patients without endometriosis-related symptoms. Group of "presence" are patients with severe and disabling symptoms endometriosis-related symptoms. ORc - *Odds Ratio* crude; ORa - *Odds Ratio* adjusted; CI - Confidence interval; Bold represents significant values;

* Pearson's chi-square test;

** Reference.

*** Missing - 6 patients did not answer whether they had dyspareunia

## Discussion

Endometriosis pain is a very common and extremely disabling disorder for women, and it is still poorly understood.^(1)^ Leptin, with its pro-inflammatory and neoangiogenic action, may play a role in the origin of the pain associated with the disease.^(2,3)^ To date, there are no biochemical and genetic markers considered efficient and accurate to assist in the individualized treatment of these women, and this is a broad field of research that may provide new diagnostic and therapeutic strategies. Therefore, in the present study, the association of the *LEPR* rs1137100 A>G SNPs with the prevalence of painful symptoms of 237 women with endometriosis in the Brazilian population was explored.

The variant allele frequencies of *LEP* rs7799039 A and *LEPR* rs1137100 G found in this study were similar to previous findings for healthy Brazilians.^(8)^ To date, there are no data in the literature on the association of these SNPs with the development of endometriosis, and further studies are needed in different populations.

The neurotropic and neuroprotective activity of cytokines suggests that inflammation is a major cause of pain associated with endometriosis.^(9)^ In turn, leptin is one of these cytokines that has already been reported as a hormone capable of inducing inflammatory reactions and immune cell-mediated modulation.^(9)^ Moreover, leptin deficiency increases susceptibility to infectious stimuli and is associated with dysregulation of cytokine production.^(10)^ Endometriosis pain can initially be understood as nociceptive inflammatory pain, since relevant high levels of pro-inflammatory factors, such as IL-6 and -8 and prostaglandin E2 (PGE2), have been demonstrated in women with the disease. These inflammatory mediators activate visceral and peritoneal nerve fibers, leading to increased sensitivity to pain. Inflammation and cellular damage cause pain, reducing as the reaction slows down, leading to the hypothesis that leptin may also be related to these inflammatory modulations.^(11)^ Furthermore, it has been observed that women with chronic pelvic pain related to endometriosis have increased leptin levels.^(12)^

The *LEPR* rs1137100 SNP, which was associated with pain in the present study, is a missense variant, located in exon 4, being a point mutation in which a single nucleotide changes results in a codon that encodes a different amino acid (lysine to arginine), which may influence the structure and function of LEPR.^(4,5)^ The leptin receptors (Ob-R) on the cell membrane or in the cytoplasm of both normal and tumor endometrial cells alter the action of leptin on the proliferation and invasion of these cells, depending on the dose and the rate of receptor expression.^(13)^ Consequently, the presence of the *LEPR* rs1137100 SNP can impact the binding capacity of leptin to its receptor, influencing conditions such as obesity, type 2 diabetes, cancers, and inflammatory diseases^(13-16)^ A genome-wide association study carried out in the European population with 11,504 women associated increased plasma levels of the leptin receptor with the presence of 132 SNPs of the *LEPR* gene, including rs2767485, rs1751492, rs4655555, rs1137101 and our rs1137100 SNP, which remained significant even after adjustment for confounding factors.^(17)^ Therefore, based on the results of this study, the possible function of the SNP and the relationship of leptin with the symptoms of endometriosis, it is suggested that the presence of the *LEPR* rs1137100 G allele may alter the conformation of the leptin receptor,^(4,5)^ facilitating its binding with leptin and potentially increasing expression levels, although no studies have directly demonstrated this effect. This can modulate the inflammatory response present in endometriosis foci, generating or intensifying the pain present in this disease.^(12)^

The *LEP* rs7799039 SNP is located in the promoter region of the gene on chromosome 7 and has been associated with leptin expression and secretion.^(18-20)^ According to the study carried out by Hoffstedt et al. in 2002, individuals with the AA genotype for this SNP have serum leptin levels approximately 50% higher than individuals with the GA+GG genotypes, with significance maintained with the analysis adjusted for BMI.^(18)^ Although the present study did not find a significant association of *LEP* rs7799039 in the symptoms of endometriosis, several other studies have positively associated this SNP with other metabolic and inflammatory diseases.^(21-24)^ The small sample size, the type of population studied, the multifactorial influence of the disease, as well as the individual analysis of the polymorphism may have impacted the statistical power of the study to detect true associations.^(25)^ Therefore, studies that evaluate the combination of other *LEP* polymorphisms should still be analyzed.

This study presents some strengths that must be taken into consideration: (i) to our knowledge, this was the first study that determined the allelic and genotypic frequencies of the *LEP* rs7799039 and *LEPR* rs1137100 SNPs in women with endometriosis; (ii) it was the first study that evaluated the association of these SNPs with disease symptoms; (iii) the study population was entirely composed of Brazilian women recruited from public reference hospitals for the diagnosis of endometriosis; and (iv) the endometriosis cases were surgically diagnosed with histopathological confirmation or MRI for deep endometriosis, which is a sensitive and specific diagnostic method.^(26)^ However, as in any retrospective observational study, there is the likelihood of recall bias at the time of recruitment, in addition to women with mild and moderate symptoms of endometriosis not being included and the lack of information on the duration of symptoms, which may have biased the study. Another limitation, leptin levels and LEPR expression in women with endometriosis and in different genotypes could not be directly determined due to the lack of an adequate sample for measurement.

The present study provides a clue for future investigations to understand the contribution of genetic polymorphisms of the leptin receptor in the genetic predisposition to endometriosis-related symptoms. Finally, it can help to explore therapeutic options for the painful symptoms present in women with endometriosis, and thus contributing to improving the quality of life of these women.

## Conclusion

In conclusion, the *LEPR* rs1137100 SNP may be associated with greater susceptibility to chronic pelvic pain and dyspareunia in women with endometriosis.
